# Genome-wide characterization of the soybean DOMAIN OF UNKNOWN FUNCTION 679 membrane protein gene family highlights their potential involvement in growth and stress response

**DOI:** 10.3389/fpls.2023.1216082

**Published:** 2023-09-08

**Authors:** Bhagwat Nawade, Tejas C. Bosamia, Jae Hyun Lee, Jin Hoon Jang, Ok Ran Lee

**Affiliations:** ^1^ Department of Applied Plant Science, College of Agriculture and Life Sciences, Chonnam National University, Gwangju, Republic of Korea; ^2^ Interdisciplinary Program in IT-Bio Convergence System, Chonnam National University, Gwangju, Republic of Korea; ^3^ Plant Omics Division, Council of Scientific and Industrial Research-Central Salt and Marine Chemical Research Institute (CSIR-CSMCRI), Bhavnagar, Gujarat, India

**Keywords:** DUF679, soybean, abiotic stress, genomic analysis, cis-acting elements

## Abstract

The *DMP* (DUF679 membrane proteins) family is a plant-specific gene family that encodes membrane proteins. The DMP family genes are suggested to be involved in various programmed cell death processes and gamete fusion during double fertilization in *Arabidopsis.* However, their functional relevance in other crops remains unknown. This study identified 14 genes from the DMP family in soybean (*Glycine max*) and characterized their physiochemical properties, subcellular location, gene structure, and promoter regions using bioinformatics tools. Additionally, their tissue-specific and stress-responsive expressions were analyzed using publicly available transcriptome data. Phylogenetic analysis of 198 *DMP*s from monocots and dicots revealed six clades, with clade-I encoding senescence-related *AtDMP1/2* orthologues and clade-II including pollen-specific *AtDMP8/9* orthologues. The largest clade, clade-III, predominantly included monocot *DMP*s, while monocot- and dicot-specific *DMP*s were assembled in clade-IV and clade-VI, respectively. Evolutionary analysis suggests that soybean *GmDMP*s underwent purifying selection during evolution. Using 68 transcriptome datasets, expression profiling revealed expression in diverse tissues and distinct responses to abiotic and biotic stresses. The genes *Glyma.09G237500* and *Glyma.18G098300* showed pistil-abundant expression by qPCR, suggesting they could be potential targets for female organ-mediated haploid induction. Furthermore, cis-acting regulatory elements primarily related to stress-, hormone-, and light-induced pathways regulate *GmDMP*s, which is consistent with their divergent expression and suggests involvement in growth and stress responses. Overall, our study provides a comprehensive report on the soybean *GmDMP* family and a framework for further biological functional analysis of *DMP* genes in soybean or other crops.

## 1 Introduction

The DMPs (DUF679 membrane proteins) are membrane proteins found exclusively in green plants (Viridiplantae) and especially in flowering plants (Cyprys et al., 2019). The members of this uncharacterized plant-specific gene family are predicted to be involved in various physiological processes, particularly senescence and reproduction (Kasaras and Kunze, 2010; Cyprys et al., 2019; Zhu et al., 2021). The DMPs are integral membrane proteins with no sequence similarity to functionally assigned protein domains, channels, transporters, or any other membrane proteins in any kingdom (Kasaras and Kunze, 2010). A total of ten members, located across four chromosomes, were identified in the *DMP* gene family in *Arabidopsis* (Kasaras and Kunze, 2010). The *Arabidopsis* DMP family proteins have four transmembrane spans and amino- and carboxy-termini in the cytosol (Kasaras and Kunze, 2010). Among 10 *AtDMP*s, *AtDMP1* was reported as a senescence-associated gene, as it was upregulated during natural or developmental senescence of siliques, rosettes, and cauline leaves, as well as in dark-induced senescence of attached and detached leaves (Van der Graaff et al., 2006). Additionally, *AtDMP1* is highly expressed in dehiscence and abscission zones of siliques (Kasaras and Kunze, 2010). Furthermore, *AtDMP1*, with dual endoplasmic reticulum (ER) and tonoplast localization, is engaged in membrane fission during the breakdown of these organelles during leaf senescence as well as membrane fusion during root vacuole biogenesis (Kasaras et al., 2012). Like *AtDMP1*, *AtDMP3* and *AtDMP4* were upregulated in senescing rosette leaves, cauline leaves, and siliques, indicating overlapping functions during senescence. Additionally, *AtDMP3* and *AtDMP4* transcripts were detected in roots and flowers, respectively. Three *AtDMPs*, *AtDMP2*, *AtDMP6*, and *AtDMP7*, were expressed in all organs (Kasaras and Kunze, 2010). *AtDMP4* was coregulated with the core of dPCD (developmental-programmed cell death) marker genes, including BIFUNCTIONAL NUCLEASE1 (*BFN1*), PUTATIVE ASPARTIC PROTEASE A3 (*PASPA3*), RIBONUCLEASE3 (*RNS3*), CYSTEINE ENDOPEPDITASE 1 (*CEP1*), and EXITUS1 (*EXI1*). They were commonly upregulated in differentiation-induced and age-induced dPCD types (Olvera-Carrillo et al., 2015). Additionally, during *Arabidopsis* stigma senescence, *AtDMP4*, along with *BFN1, RNS3, EXI1, CEP1, DMP4*, and *PASPA3* were reported to copromote senescence and dPCD (Gao et al., 2018). Furthermore, a comprehensive genome-wide analysis of four cotton species (*Gossypium arboreum*, *G. raimondii*, *G. hirsutum*, and *G. barbadense*) identified a total of 58 *DMPs*. Analysis of the expression patterns of these DMPs unveiled their possible involvement in key biological processes, such as plant senescence, flower reproductive development, and stress response (Zhu et al., 2021).

The sperm-specific *AtDMP8* and *AtDMP9* are reported to involve gamete fusion with a more significant effect on sperm-egg fusion (Takahashi et al., 2018; Cyprys et al., 2019). Moreover, the detection of a mutation in the haploid inducer-associated locus *qhir8* (specifically the *ZmDMP* gene) in maize and loss-of-function mutations in the *Arabidopsis AtDMP8* and *AtDMP9* led to the development of an effective haploid induction system in dicots (Zhong et al., 2019; Zhong et al., 2020). Although *AtDMP8* and *AtDMP9* orthologues of the *DMP* gene family are utilized to induce maternal haploids in dicots (Zhong et al., 2020; Wang et al., 2022; Zhong et al., 2022a; Zhong et al., 2022b), information about other members in the family and their functional relevance needs to be investigated in crops. Soybean (*Glycine max*), the most widely grown commercial legume and oilseed crop, is commonly used for human consumption, livestock feed, oil production (Hartman et al., 2011). Soybeans contributed 70.39% of plant-based protein meals and 28.55% of plant-based oils in 2020/2021 globally (Market View Data Base, United Soybean Board 2021, accessed on 26 July 2022) and have been referred to as a ‘Wonder crop’ and the ‘Golden bean’ for their high nutritional content, oil content (18%), high-quality proteins (40%), high productivity, versatile uses, and profitability (Rajendran et al., 2022). Being a major oilseed crop, soybean has gained increasing attention in the genomics area, and the availability of genome sequence information accelerated the characterization of important gene families in soybean (Zhang et al., 2022a). However, studies on soybean *DMPs* investigating their genome-wide occurrence, phylogenetics, and functions are still lacking.

CRISPR (clustered regularly interspaced short palindromic repeats)/Cas9 technology relies on the precise and efficient introduction of double-stranded DNA breaks by the Cas9 nuclease, guided by a sgRNA. Predicting the cleavage efficacy of sgRNAs *in vitro* prior to their use in plant genome editing offers time, labor, and cost savings by enabling the selection of efficient sgRNAs, optimizing experimental design, and enhancing the success rate and accuracy of editing experiments (Mehravar et al., 2019; Bente et al., 2020). The integration of preassembled Cas9 enzyme with *in vitro*-transcribed sgRNA, known as the ribonucleoprotein complex (RNP), has been successfully employed in various plant species such as soybean (Kim et al., 2017; Subburaj et al., 2022), wheat (Liang et al., 2017), *Brassica* (Murovec et al., 2018; Jeong et al., 2019), maize (Sant’Ana et al., 2020), grapevine (Malnoy et al., 2016), apple (Malnoy et al., 2016), and pepper (Kim et al., 2020). This approach is used to assess the *in vitro* cleavage efficiency sgRNAs and their suitability for subsequent genome editing experiments.

In this study, we identified a total of 198 *DMP* genes from monocot and dicot crops and analyzed their phylogenetic relationships. Further, we comprehensively analyzed the physiochemical properties, subcellular location, gene structures, motifs, and promoters of the *GmDMP* family using bioinformatics tools. In order to understand the age- and tissue-specific expression levels of *DMP*s, as well as their responses to different stresses, publicly available transcriptome data were processed for analysis. Additionally, we conducted real-time quantitative PCR (qPCR) analysis specifically on floral and vegetative tissues to gain further insights into their expression profiles. Subsequently, based on floral expression pattern, we selected two genes with potential applications in haploid induction and conducted an *in vitro* cleavage assay to assess the cleavage efficiency of the selected sgRNAs. Our findings consolidate the information available in public databases on the *DMP* gene family and provide a comparative account of *DMPs* from soybean that would be useful for subsequent functional characterization. This study also identified potential candidate genes associated with haploid induction, growth, and stress response, which have the potential to accelerate soybean breeding.

## 2 Materials and methods

### 2.1 Identification and retrieval of *DMP* genes from monocot and dicot plants

Protein sequences of *Arabidopsis DMP*s were retrieved from Phytozome (https://phytozome-next.jgi.doe.gov/) and used as queries to perform blastp searches within the genome sequences of *Glycine max* (Wm82.a2.v1), acquired from the Soybase database (https://soybase.org/soyseq/); *Arachis hypogea* (v1), acquired from the Peanutbase database (https://peanutbase.org/); *Gossypium hirsutum* (v1.1), *Helianthus annuus* (r1.2), *Linum usitatissimum* (v1.0), *Setaria italica* (v2.2), *Medicago truncatula* (Mt4.0v1), *Oryza sativa* (v7.0), *Solanum lycopersicum* (ITAG2.4), *Sorghum bicolor* (BTx642 v1.1), and *Zea mays* (RefGen_V4), acquired from the Phytozome database (https://phytozome-next.jgi.doe.gov/); *Sesamum indicum*, obtained from the Ensembl Plants database (http://plants.ensembl.org/index.html); and *Brassica napus* obtained from the Brassicaceae Database (BRAD, http://brassicadb.cn/#/). All were accessed in April 2022. Subsequently, retrieved gene sequences were verified for the DMP domain (IPR007770) using the National Center for Biotechnology Information (NCBI) Conserved Domain Database (https://www.ncbi.nlm.nih.gov/cdd) (Marchler-Bauer et al., 2010), Simple Modular Architecture Research Tool (SMART; http://smart.embl.de/) (Letunic and Bork, 2018), and the Pfam tool (http://pfam.xfam.org/) (El-Gebali et al., 2019). All verification databases and tools were accessed in May 2022.

### 2.2 Protein sequence alignment and phylogenetic analysis

The full-length amino acid sequences of DMP family members from different oilseed crops—including *Glycine max, Arachis hypogea, Brassica napus, Gossypium hirsutum*, *Helianthus annuus, Linum usitatissimum*, and *Sesamum indicum*—were aligned using the ClustalW program with the default parameters, and a phylogenetic tree was constructed using the neighbor-joining (NJ) method, both in the MEGA software suite (version 11.0) (Tamura et al., 2021). The phylogenetic analysis was performed using the p-distance model, pairwise deletion, and 1,000 bootstrap replicates. Then, the iTOL tool (https://itol.embl.de/) was used to visualize the unrooted phylogenetic tree (Letunic and Bork, 2021). The accession numbers of the DMPs used for the phylogenetic analysis are listed in **Supplementary Table S1**.

### 2.3 Physiochemical properties and subcellular location prediction

The physicochemical properties, *viz.*, molecular weight (Mw), theoretical isoelectric point (pI), instability index, grand average of hydropathicity (GRAVY), and aliphatic index of soybean DMP proteins were predicted using the ProtParam tool on the ExPASy server (https://web.expasy.org/protparam/) (Gasteiger et al., 2005). The subcellular location of DMP proteins was predicted using DeepLoc 2.0 (https://services.healthtech.dtu.dk/service.php?DeepLoc-2.0) (Thumuluri et al., 2022). The numbers of transmembrane domains in soybean DMP proteins were predicted using the DeepTMHMM tool (https://dtu.biolib.com/DeepTMHMM) (Hallgren et al., 2022).

### 2.4 Conserved motif and intron/exon structure analysis

The conserved motifs were predicted by the MEME (Multiple Expectation Maximization for Motif Elicitation) tool (http://meme-suite.org/tools/meme), an online program for motif discovery (Bailey et al., 2006). Using MEME suite (Version 5.4.1), the motifs were searched with these parameters: the ‘motif discovery mode’ was set to ‘classic mode’, the ‘site distribution’ to ‘zero or one occurrence per sequence’, the ‘number of motifs’ to 10, the width of motifs was set to between 6 and 50, and the required number of sites for each motif to between 2 and 600. The exon-intron structures of the *DMP* genes were analyzed using the Gene Structure Display Server (GSDS v2.0, http://gsds.gao-lab.org/) (Hu et al., 2015). TBtools software was employed to visualize the distribution of the motif along with the phylogenetic tree and gene structures (Chen et al., 2020).

### 2.5 Analysis of promoter regions

The 2000 bp sequences upstream of the start codon for each *DMP* gene were retrieved from the soybean and *Arabidopsis* database from Phytozome (https://phytozome-next.jgi.doe.gov/). The sequences were submitted to the CARE (Cis-Acting Regulatory Element) search tool (https://bioinformatics.psb.ugent.be/webtools/plantcare/html/) in the PlantCARE database (Lescot et al., 2002) to predict and analyze the cis-acting elements related to plant growth and development, hormones, and stress and light responses in the promotor regions of soybean *DMP* genes.

### 2.6 Selection pressure and duplicated gene pairs analysis

The Ka/Ks ratio was used to assess selection history and divergence time. The number of synonymous (Ks) and nonsynonymous (Ka) substitutions of duplicated *DMP* genes were computed using the Ka/Ks Calculator tool (http://services.cbu.uib.no/tools/kaks). The divergence time (T) was calculated using the formula T=Ks/(2× 6. 1× 10^−9^) ×10^−6^ million years ago (Mya) (Kim et al., 2013).

### 2.7 *In silico* expression analyzes of soybean DMPs in various tissues and developmental stages

To understand the differential expression patterns of soybean DMPs during plant growth and senescence and determine their responses to different stresses, a total of 68 SRA (Sequence Read Archive) datasets comprised of 12 different treatments were downloaded from the NCBI database (**Supplementary Table S2**). The raw reads were first analyzed using FastQC (www.bioinformatics.babraham.ac.uk/projects/fastqc), and then low-quality bases (< 30 phred score), low-complexity and short sequences (< 50bp), and adapter sequences were removed using an in-house perl script and TrimGalore v0.6.5 (Krueger, 2015). The remaining high-quality reads were used to further analyze transcript abundance using the Galaxy platform. The high-quality reads from each dataset were mapped to the soybean genome (Gmax_275_v2.0.fa, downloaded on 17.07.2022) using the HiSAT2 aligner with the default parameters (Kim et al., 2015). The mapped reads for each sample were counted (‘wcountedount’) using StringTie v1.2.0 (Pertea et al., 2015), and the abundance of genes and transcripts, in fragments per kilobase of transcript per million mapped reads (FPKM), were computed from those mapped to the *Glycine max* genome annotation. Furthermore, to compare the normalized read count data (FPKM) across the different tissues and treatments, the Z-score was calculated on a gene-by-gene basis by subtracting the overall mean and then dividing by standard deviation. A heatmap was constructed using Multi Experiment Viewer (MeV) v4.9.0. Additionally, publicly available expression data for 65 anatomical parts and seven developmental stages housed in the Genevestigator database (www.genevestigator.com) (Hruz et al., 2008) were retrieved using the Phytozome IDs for soybean.

### 2.8 Quantitative real-time PCR analysis

Seeds of the soybean cultivar Williams 82 were germinated and cultivated under controlled conditions in a growth chamber, maintaining a temperature of 25 ± 1°C and a 16 h/8 h light/dark cycle. After 60 days of growth, total RNA was extracted from different tissues of the plants, including leaves, stems, flower buds, sepals, petals, pistils, and pollen. To ensure precise sampling, we collected three newly formed leaves from the top of the plants. Stems were sampled 3 cm below the uppermost shoot apex. To obtain sufficient cDNA for qPCR experiments, floral organs were collected from four plants (25 open flowers per plant). Thus, in total, 100 open flowers were pooled in a single biological sample representing four plants. RNA was extracted from pooled samples with subsequent construction of the corresponding cDNA samples. Therefore, the qPCR analysis involved three technical replicates and pooled single biological replicate from four plants. Pooling samples from several independent plants is a common practice in gene expression studies to increase sample size and statistical power while reducing inter-individual variability (Rego et al., 2019; Moebes et al., 2022). Microscopy (EZ4 HD, Leica, Wetzlar, Germany) was used for observing and sampling the floral organs. All flower organs were collected from 100 open flowers measuring approximately 2 mm in size. Sepals and petals were carefully separated from the flower using a needle. For the pistil, only those free from pollen contamination were collected by cutting them with a needle. Immediately after collection anthers were immersed in distilled water and opened using a needle to obtain pollen. Subsequently, we isolated the pollen by passing it through a 50-um nylon filter (04-0042-2317, Sysmex, Görlitz, Germany) to exclude any other tissues. Plant RNA extraction kits (Takara, Shiga, Japan) were used to extract the RNA, following the manufacturer’s instructions. The quality of RNA was checked by agarose gel electrophoresis, and its quantity was determined using a spectrophotometer (Nano-MD UV-Vis, Scinco, Seoul, Korea). For cDNA synthesis, the RevertAid Reverse Transcriptase (Thermo, Waltham, MA, USA) was used in 20 μL reaction volumes. Real-time quantitative PCR (qPCR) was performed in a Thermal Cycler Dice real-time PCR system (Takara, Shiga, Japan) using TB Green™ Premix Ex Taq™ Master Mix (Takara, Shiga, Japan). The relative expression levels of the target genes were quantified in comparison to leaf tissue using the 2^−▵▵CT^ method. Soybean actin11 (*Glyma.18G290800*) was used as the reference gene for normalization. The analysis was performed with the sample representing three technical replicates and pooled single biological sample from four plants. Gene-specific primers were designed using Primer3Plus (https://www.bioinformatics.nl/cgi-bin/primer3plus/primer3plus.cgi), and their details are listed in **Supplementary Table S3**.

### 2.9 *In vitro* cleavage assay

#### 2.9.1 Designing the single-guide RNAs

Cas-Designer, a web-based tool RGENs (http://www.rgenome.net/) (Park et al., 2015), was utilized for designing the sgRNAs against the Glycine max (Wm82.a2.v1) genome with default settings. This process resulted in the prediction of a set of candidate sgRNAs along with their respective cleavage positions, out-of-frame scores, and potential mismatches. However, it is important to note that not all sgRNAs display the same cleavage efficiency (Karmakar et al., 2021). To evaluate cleavage efficiency, two sgRNAs, namely SgGmDMP#1 (5’-GGAGGACCATCTCAAAAGTGAGG-3’) and SgGmDMP#2 (5’-CTCCATATCCTTATCCTTCCCGG-3’), were selected. An *in vitro* screening method was optimized to assess and identify efficient sgRNA.

#### 2.9.2 *In vitro* synthesis of SgRNAs

The SgRNAs were transcribed *in vitro* using the GeneArt™ Precision gRNA Synthesis Kit (Invitrogen, USA) following the manufacturer’s protocol. Briefly, the transcription templates were prepared by PCR assembly of the gRNA-DNA template using synthetic forward and reverse oligonucleotides with the Tracer Fragment + T7 Primer Mix (**Supplementary Table S3**). The resulting PCR product containing a T7 promoter sequence (5’-TAATACGACTCACTATA-3’) and the sgRNA sequence without the PAM region was purified using the gel-purification kit (GeneAll, Seoul, Korea). The *in vitro* transcription reaction was conducted with a total volume of 20 μL, consisting of 6 μL of purified gRNA-DNA template, 8 μL of NTP mix (25 mM each NTP), 4 μL of 5X TranscriptAid™ reaction buffer, and 2 μL of TranscriptAid™ enzyme mix. The reaction was incubated at 37°C for 3 h. Following the transcription reaction, the synthesized sgRNAs were treated with DNAase to remove any residual DNA and purified using the gRNA Clean Up Kit (Invitrogen, USA). The concentration of the purified sgRNA was determined using a UV spectrophotometer (Nano-MD UV-Vis, Scinco, Seoul, Korea). The sgRNA samples were then diluted to a final concentration of 1 μM and stored at -80°C for future use.

#### 2.9.3 *In vitro* Cas9-cleavage assay of PCR products

To generate DNA templates containing the sgRNA target sites, a PCR amplification step was performed using soybean genomic DNA and flaking primers (**Supplementary Table S3**). The resulting PCR products were gel purified and quantified before stored in aliquots at −20°C. Cas9 cleavage reactions (final volume 15 µl) were assembled by combining: 1x Orange Buffer (O-buffer, Thermo Fisher Scientific), 300 ng sgRNA, 250 ng of recombinant *Streptococcus pyogenes* Cas9-NLS protein (TrueCut™ Cas9 Protein v2, Invitrogen), and ddH_2_O to reach a final volume of 15 µL. The mixtures were incubated for 10 mins at 22°C to allow the formation of the ribonucleoprotein (RNP) complex. Subsequently, 250 ng of template DNA was added, and the reactions were incubated for 2 h at 37°C. After incubation, 1.2 µL of RNase A (5 µg) and 1.2 µL of Proteinase K (2 mg/mL) were added to the reaction, followed by incubation at 37°C for 20 min. Finally, the reactions were heat-inactivated at 80°C for 10 min. The products of each reaction were analyzed by electrophoresis on a 2% agarose gel.

## 3 Results

### 3.1 Identification of *DMP* genes in soybean and other crops

The genomes of monocot and dicot plants were mined for *DMP* genes using the Pfam database-derived HMM profile of the DMP domain (PF05078) as the query. We retrieved 198 putative *DMP* genes after verification. Among them, we detected 14 *DMP* genes in soybean, 14 in peanut, 7 in tomato, 14 in foxtail millet, 19 in rice, and 16 in sorghum. The *DMP* gene names, locus IDs, and other features are shown in **Supplementary Table S1**. The four monocots, foxtail millet, sorghum, maize, and rice, contained 11–16 *DMP* homologues, whereas the dicots contained from 5 (*M. truncatula*) to 14 (*G. max*) homologues.

### 3.2 Phylogenetic analysis

**Figure 1:**
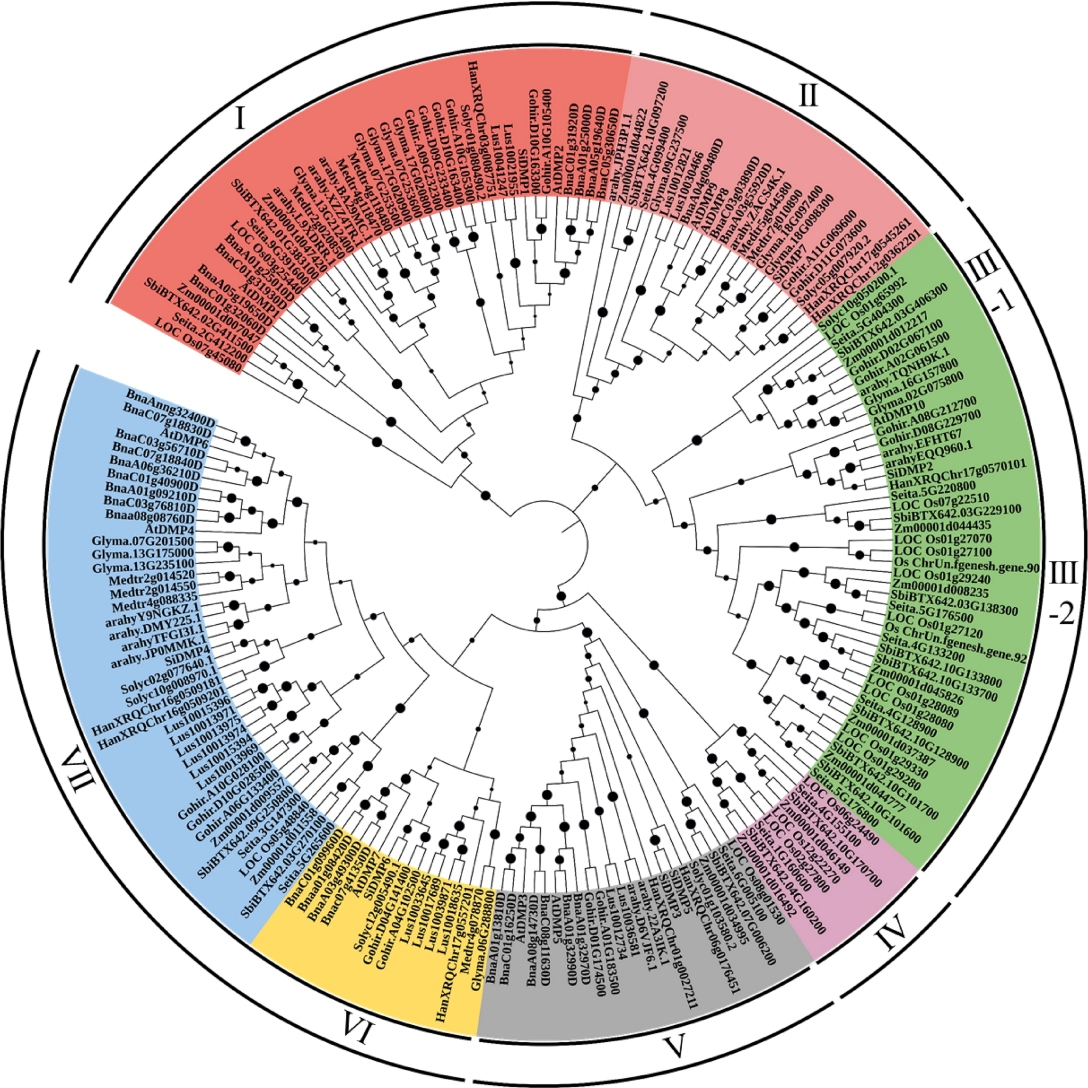
Phylogenetic relationships of the DMP proteins identified from monocot and dicot crops constructed using the N-J method with 1000 bootstraps in MEGAv11. Bootstrap values are shown on the nodes. DMP proteins were grouped into seven clades, which are denoted by color. For accession ID details of the genes, refer to **Supplementary Table S1**.

To analyze the phylogenetic relationships of the DMP family, an unrooted phylogenetic tree was constructed using the 198 DMP proteins of monocot and dicot crops (**Figure 1**). The DMP proteins were clustered into seven major clades. Clade-I, with 40 DMPs, encoded senescence-related AtDMP1 and AtDMP2 orthologues, while the pollen-specific AtDMP8 and AtDMP9 orthologous proteins (23 DMPs) were grouped into clade II, which included three soybean DMPs: *Glyma.09G237500, Glyma.18G097400*, and *Glyma.18G098300*. Clade-III was the largest clade with 45 DMPs predominated by 28 monocot DMPs, which clustered to form sub-clade-III-2. Two soybean DMPs, *Glyma.16G157800* and *Glyma.02G075800*, formed sub-group III-1 along with orthologues of AtDMP10. Notably, clade-IV, containing nine DMPs, and clade-VI, containing 16, were monocot and dicot specific. Interestingly, group V, comprising AtDMP3 and AtDMP5, did not contain any soybean DMP proteins. It is possible that the region containing these orthologues was deleted during the evolution of the soybean genome. Dicot-predominant clade-VII, with 42 DMPs, harbored AtDMP4, and AtDMP6 orthologues, including three soybean DMPs: *Glyma.07G201500*, *Glyma.13G175000*, and *Glyma.13G235100*.

### 3.3 Physicochemical properties of soybean DMP proteins

The physicochemical properties of soybean DMP proteins were analyzed (**Table 1**). The GmDMP proteins varied in length, molecular weight, theoretical isoelectric point, aliphatic index, and other properties. The *GmDMP*s were predicted to encode polypeptides from 136 to 222 amino acids in length, with predicted molecular weights ranging from 15.37 to 25.07 kD. The predicted aliphatic indices ranged from 62.35 to 108.63. The aliphatic index indicates the thermostability and half-life of a protein (Kyte and Doolittle, 1982). The theoretical isoelectric points (pIs) ranged from 4.86 (*Glyma.13G235100*) to 9.74 (*Glyma.09G237500*), and the grand average of the hydropathicity values of all GmDMP proteins was positive except *Glyma.09G237500*, indicating their transmembrane nature. The subcellular localization was predicted using DeepLoc 2.0, and GmDMPs were found to be located on various parts of different cell organelles, including one in the nucleus, six in the ER, five in the lysosome/vacuole and two in ER, lysosome/vacuole both. All GmDMP proteins, except *Glyma.09G237500*, are predicted to have four transmembrane helix domains.

**Table 1:** *In silico* prediction of the physiochemical properties of soybean DMPs.

Gene IDs	Chromosome location	Length (aa^a^)	Mol. Wt.^b^ (kDa)	Theoretical pI	Instability index	Stable yes/no	Aliphatic index	GRAVY^c^	TMDs^d^	Subcellular location
*Glyma.17G020800*	Chr17	175	20.26	9.27	35.56	stable	108.63	0.381	4	ER^e^
*Glyma.17G020900*	Chr17	209	22.41	6.81	27.15	stable	83.92	0.054	4	ER
*Glyma.09G237500*	Chr09	136	15.37	9.74	63.14	unstable	62.35	-0.404	0	Nucleus
*Glyma.07G253600*	Chr07	220	23.43	6.41	34.77	stable	86.77	0.111	4	ER, Lysosome/Vacuole
*Glyma.07G253500*	Chr07	222	23.59	6.01	34.36	stable	83.83	0.082	4	ER
*Glyma.07G201500*	Chr07	208	23.39	5.58	41.17	unstable	92.74	0.201	4	ER
*Glyma.13G235100*	Chr13	211	22.88	4.86	51.11	unstable	96.16	0.28	4	Lysosome/Vacuole
*Glyma.13G212400*	Chr13	204	21.99	8.69	39.18	stable	79.75	0.205	4	ER, Lysosome/Vacuole
*Glyma.13G175000*	Chr13	217	24.02	5.91	45.07	unstable	92.07	0.306	4	Lysosome/Vacuole
*Glyma.02G075800*	Chr02	218	24.95	5.63	53.16	unstable	94.77	0.097	4	Lysosome/Vacuole
*Glyma.06G288800*	Chr06	207	22.96	9.13	27.93	stable	101.21	0.292	4	Lysosome/Vacuole
*Glyma.18G098300*	Chr18	214	23.73	8.22	32.14	stable	84.67	0.175	4	ER
*Glyma.18G097400*	Chr18	214	23.75	8.22	28.04	stable	83.27	0.179	4	ER
*Glyma.16G157800*	Chr16	219	25.07	5.8	55.1	unstable	93.42	0.104	4	Lysosome/Vacuole

a amino acids.

b Molecular weight.

c Grand average of hydropathicity.

d transmembrane domains.

e endoplasmic reticulum.

### 3.4 Exon-intron structure, motif, and sequence analysis

**Figure 2:**
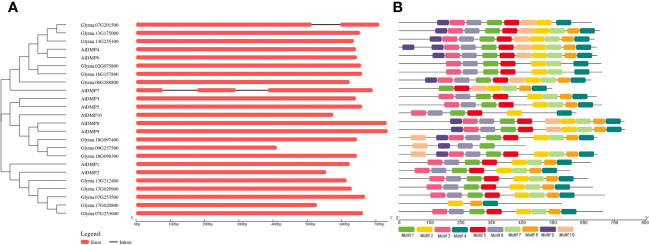
Genomic structure and motif composition comparisons between *Arabidopsis* and soybean *DMP* genes. **(A)** Phylogenetic tree and genomic structures of the genes. Exons and introns are indicated with boxes and black lines, respectively. **(B)** Motif composition of the *DMP* genes. Conserved motifs are indicated by colored boxes. For the details of each motif, refer to **Supplementary Table S4**.

The exon and intron arrangement among the *GmDMP* gene family members was analyzed using the GSDS web server, revealing that, except for *Glyma.07G201500*, none possessed any introns. All gene structures of the *DMP* family had only one exon without an intron and with a conserved domain. To understand the diversity and similarity of gene structure and motif among the *Arabidopsis* and soybean proteins, we constructed a separate phylogenetic tree using GSDS (**Figure 2**). Among the ten motifs identified, five motifs encoded a DMP domain, and all DMP proteins have motifs ranging from 3 to 10 (**Figure 2B**, **Supplementary Table S4**). Members of the same group shared a somewhat common motif distribution pattern, suggesting their similar functional relevance and conserved protein architecture. Some motifs were absent in specific groups. For example, motifs 9 and 10 were absent in all the members of the clade I proteins, and motifs 8, 9, and 10 were absent in the members of subclade III-1. The structure and motif conservation within groups support the results of the phylogenetic analysis.

### 3.5 Ka/Ks selective pressure analysis of soybean *DMP* gene family

The substitution rates among the duplicated gene pairs were less than 1 (**Table 2**), signifying a strong purifying selective pressure during the evolution of the soybean *DMP* gene family. In addition, the approximate time of the duplication events were calculated to determine the extent and nature of selection pressure in *GmDMP* genes. The five *GmDMP* pairs were duplicated from 2.580 Mya (*Glyma.18G097400* and *Glyma.18G098300*) to 36.533 Mya (*Glyma.13G235100* and *Glyma.06G288800*) (**Table 2**). The soybean genome is reported to have experienced two rounds of whole genome duplication (WGD), the first duplication occurred prior to the divergence of legume subfamily Papilionoideae (58–60 Mya) and a recent Glycine-specific WGD occurred approximately 13 Mya (Schmutz et al., 2010). Among the six *DMP* duplicate gene pairs, three pairs were derived after the first WGD and three after the second WGD, including *Glyma.07G253500* and *Glyma.07G253600*, *Glyma.17G020900* and *Glyma.09G237500*, and *Glyma.13G235100* and *Glyma.06G288800*.

**Table 2:** Divergence between *DMP* gene pairs in soybean.

Gene pair		Ka^a^	Ks^b^	Ka/Ks ratio	Time (MYA^c^)
*Glyma.07G253500*	*Glyma.07G253600*	0.082	0.160	0.515	13.098
*Glyma.18G097400*	*Glyma.18G098300*	0.006	0.031	0.186	2.580
*Glyma.17G020900*	*Glyma.09G237500*	0.231	0.313	0.739	25.652
*Glyma.16G157800*	*Glyma.02G075800*	0.010	0.043	0.235	3.499
*Glyma.13G175000*	*Glyma.07G201500*	0.076	0.148	0.516	12.127
*Glyma.13G235100*	*Glyma.06G288800*	0.179	0.446	0.402	36.533

a nonsynonymous substitution rate.

b synonymous substitution rate.

c millions of years ago.

### 3.6 Cis-element analysis of *GmDMP* gene promotors

**Figure 3:**
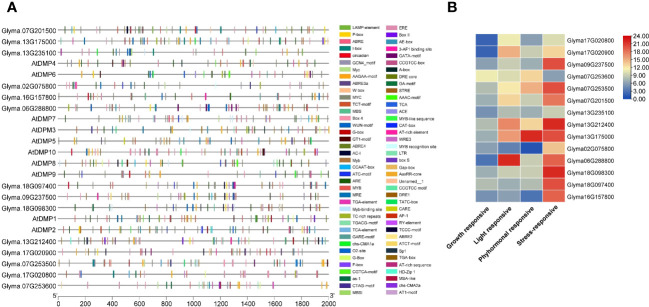
Cis-element analysis of the promoter regions of the DMP family. **(A)** Variation in different types of cis-acting regulatory elements (CAREs). **(B)** Graphical representation of CAREs. Different colored shapes represent the different elements. For details of each CARE binding site, refer to **Supplementary Table S5**.

The PlantCARE database-based analysis of the sequences upstream (2 kb) from the start codon of the *DMP* genes identified 56 CAREs (cis-acting regulatory elements; **Supplementary Table S5**). These CAREs are grouped into different categories based on their functional relevance, viz., growth and development-, light-, phytohormonal-, and stress-responsive elements (**Figure 3**; **Supplementary Table S5**). Binding sites for CAREs associated with stress responsiveness were predominant in most of the promoters; however, promoters of *Glyma.06G288800* and *Glyma.07G253600* had higher binding sites for light-responsive and phytohormonal-responsive elements (**Figure 3B**). The highest number of binding sites were detected in the *Glyma.13G175000* gene promoter (65 sites) followed by *Glyma.13G212400* (60 sites). In comparison, the lowest number of binding sites were identified in the *Glyma.02G075800* promoter (25 sites). A higher availability of stress-responsive elements in promoters suggests that their expression is linked to and regulated by stressors. The detected stress-related CAREs included dehydration-responsive (Myeloblastosis-MYB, Myelocytometosis-MYC, and Myb binding site-MBS), low-temperature-responsive (LTR), defense- and stress-responsive (TC-rich repeats), elicitor-mediated activation (AT-rich sequence), anaerobic induction (ARE), fungal elicitor-responsive (W box), and wound-responsive (WRE3) elements.

In the phytohormonal-responsive groups, sites for ABRE (abscisic acid responsiveness element) were observed in all *GmDMP* genes except *Glyma.02G075800* and *Glyma.16G157800*, while MeJA-responsive motifs, such as CGTCA and TGACG were found in 10 *GmDMPs*, and gibberellin-responsive motifs—such as P-box, GARE, and TATC-box—were found in 8, 6, and 1 *GmDMP*s, respectively. The auxin-responsive element TGA and salicylic acid-responsive element TCA appeared in five *GmDMP*s, and the ethylene-responsive element (ERE) in nine. The gene *Glyma.07G253500* contained all six hormone-related CAREs in its promoter, while *Glyma.02G075800* contained only salicylic acid-responsive elements. Most *GmDMP*s possessed at least three hormone-related elements, signifying their involvement in hormone metabolism processes and signal transduction networks regulating growth and developmental processes in soybean (**Figure 3B**). In addition, light-responsive elements—18 in total, including multiple copies of Box-4 and G-Box—were also found in most *GmDMP*s. Elements in this category are reported to play significant roles in light regulation and its related activities. Additionally, CAREs involved in growth and development responses—including circadian (circadian control), CCAAT-box (MYBHv1 binding site), MSA-like (cell cycle regulation), RY (seed-specific regulation), and AT-rich (binding site of AT-rich DNA binding protein) elements and the GCN4 motif (involved in endosperm expression)—were identified. These CAREs are associated with the vital processes of flowering, maturity, and seed development. Other CAREs involved in zein metabolism, flavonoid biosynthetic regulation, meristem expression, and secondary xylem development—Opaque2 (O2)-site (nine *GmDMP*s), Myb binding site-MBSI (*Glyma.13G235100*), CAT-box (three *GmDMP*s), and the AAGAA-motif (12 *GmDMP*s), respectively—were also detected (**Supplementary Table S5**). These results suggest that the *DMP* family genes may be involved in development and growth, hormone response processes, and stress responses in soybean.

### 3.7 Expression profiles in the soybean *DMP* gene family

#### 3.7.1 Tissue-specific expression patterns

**Figure 4:**
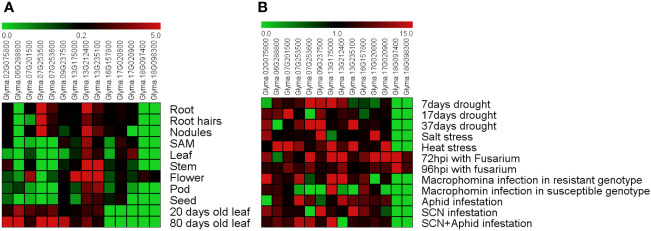
Expression pattern of soybean *DMP* genes. **(A)** Tissue- and age-dependent expression of *GmDMP* genes. **(B)** Expression profiles of *GmDMP* genes in response to abiotic and biotic stresses (SAM: shoot apical meristem, hpi: hours post-inoculation, SCN: soybean cyst nematode). Scale bar represent mean Z-score obtained from normalization of FPKM values of three replicates. Red represents high expression; green indicates low expression. For details of each gene expression, refer to **Supplementary Table S6**.

To better understand the function of the soybean *GmDMP* genes, data from publicly available transcriptome datasets and Genevestigator were used to investigate the expression profiles of *DMP* genes in various tissues under different stresses. We found that among *GmDMP* genes, *Glyma.13G212400* is highly expressed in all organs, followed by *Glyma.13G235100* and *Glyma.17G020800*. Two *GmDMP*s, *Glyma.18G097400* and *Glyma.18G098300*, were expressed only in flower tissues, while another two, *Glyma*.07G253600 and *Glyma*.07G253500, showed root-specific expression profiles (**Figure 4A**, **Supplementary Table S6**). The gene *Glyma.06G288800* was only slightly expressed in the pods. In the Genevestigator database, like transcriptome data, *Glyma.13G212400* expressed in all organs, and *Glyma.18G097400* and *Glyma.18G098300* showed anther-specific expression. Seven *GmDMPs* were mainly expressed in the anther, suggesting the role of these genes in regulating reproductive development (**Supplementary Figure S1**). To investigate the age-dependent expression of *GmDMP*s, public expression data repositories for young (20-day-old) and mature (80-day-old) leaf samples were investigated. Most of the *GmDMP* genes showed increased expression during the mature stage, including the highest increase in *Glyma.02G075800*, followed by *Glyma.09G237500, Glyma.07G253600* and then *Glyma.07G253500*. At the same time, the expression of two genes, *Glyma*.*07G201500* and *Glyma*.*13G235100*, decreased as leaves aged (**Figure 4A**, **Supplementary Table S6**).

#### 3.7.2 Expression under abiotic and biotic stresses

To explore the potential functions of soybean *DMP* genes in response to abiotic and biotic stressors, we analyzed previously reported Illumina RNA-seq data collected under drought, salt, and heat stress as well as biotic stresses including *Fusarium oxysporum, Macrophomina phasiolina*, soybean cyst nematode (SCN), and aphid infestation (**Supplementary Table S2**). The expression levels and patterns of *GmDMP*s varied considerably (**Figure 4B**, **Supplementary Table S6**). Genes *Glyma.07G253600*, *Glyma.09G237500*, and *Glyma.13G235100* had higher expression under 37 days of drought stress. Under salt stress, *Glyma.17G020800*, *Glyma.02G075800*, and *Glyma.09G237500* were highly expressed, while six genes—*Glyma.07G253500*, *Glyma.17G020900, Glyma.06G288800, Glyma.07G201500, Glyma.13G175000*, and *Glyma.13G235100*—were found to have higher transcription during heat stress. Looking at biotic stressors, *Fusarium* infection strongly upregulated the expression of *Glyma.07G253600*, *Glyma.13G212400, Glyma.17G020900*, and *Glyma.13G175000*. Interestingly, the transcripts of *Glyma.18G097400* and *Glyma.18G098300* showed a significantly increase under *Fusarium* infection, while their expression remained negligible under all other stress conditions. During *Macrophomina* infection, four genes—*Glyma.07G253500*, *Glyma.13G212400*, *Glyma.13G235100*, and *Glyma.02G075800*—were highly upregulated in resistant genotype but not in more susceptible ones, while two genes, *Glyma.17G020800* and *Glyma.13G175000*, were highly down-regulated. Under insect and nematode infestation, *DMP* genes such as *Glyma.07G253500, Glyma.07G253600, Glyma.13G212400, Glyma.02G075800, Glyma.13G175000*, and *Glyma.13G235100* showed higher transcript abundance. The differential expression patterns of soybean *DMP* genes illustrated that they play an important role in responses to diverse abiotic and biotic stressors.

#### 3.7.3 qPCR validation

**Figure 5:**
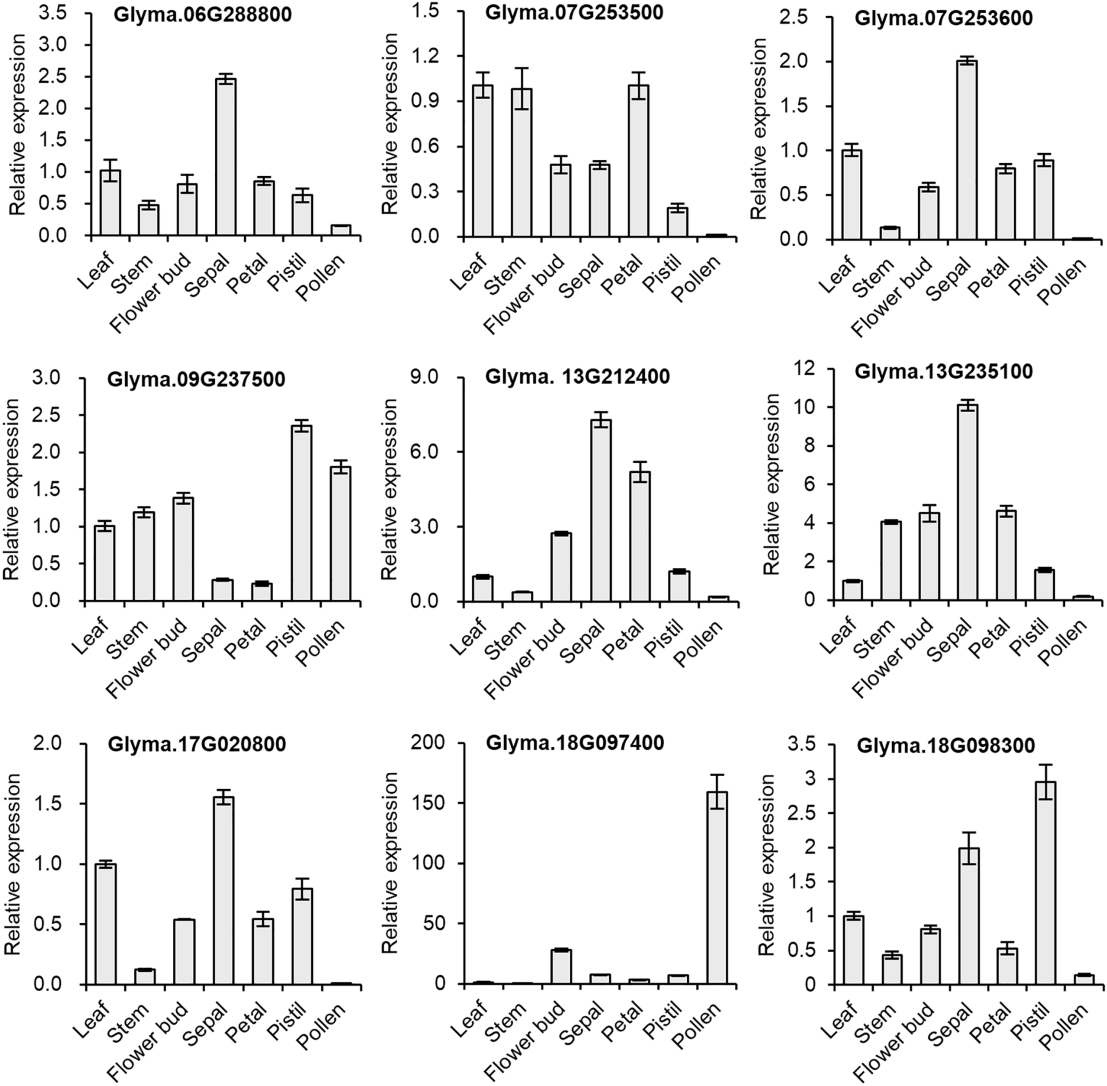
Relative expression levels of selected *GmDMP* genes in different tissues as quantified by qPCR analysis. Each data point represents the average ± SE from three independent technical replicates, obtained from pooled single biological sample from four plants.

We selected nine genes to investigate tissue-specific expression patterns at the reproductive stage (60-day-old, early flowering-stage plants) of soybean. In our qPCR analysis, we saw similar gene expression patterns to those in the *in silico* expression analysis (**Figure 5**), indicating the reliability of the computational analysis. To gain a better understanding of the expression in reproductive organs, we conducted an in-depth analysis of flower parts. Most of the *GmDMP* genes were much more highly expressed in reproductive tissues than in leaf and stem tissues. Notably, among the three *AtDMP8/9* orthologues, *Glyma.18G097400* displayed pollen-specific expression, *Glyma.18G098300* had its highest expression in the pistil, and *Glyma.09G237500* showed high expression in both pollen and pistils. In addition, *Glyma.13G212400*, *Glyma.06G288800*, *Glyma.07G253600*, *Glyma.17G020800*, and *Glyma.13G235100* exhibited their highest expression levels in sepals. Consistent with the *in silico* expression pattern, *Glyma.13G212400* showed high expression in all organs, followed by *Glyma.13G235100*. Overall, our findings provide valuable insights into the tissue-specific expression patterns of soybean *DMP* genes and highlight their potential roles in reproductive processes.

### 3.8 *In vitro* cleavage efficiency analysis

**Figure 6:**
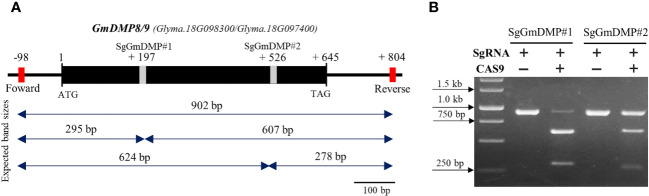
*In vitro* SgRNA cleavage efficiency analysis. **(A)** Genomic DNA structure of *GmDMP8* and *GmDMP9*. Grey boxes indicate sgRNA target sites. Expected band sizes after cleavage assay using each sgRNA were indicated by double sided arrow. **(B)** *In vitro* cleavage assay to check the efficiency of selected SgRNAs from *GmDMP8* and *GmDMP9* genes.

As the first step in identifying sgRNAs that could efficiently edit the target genes in soybean, we conducted an *in vitro* cleavage assay to assess the endonuclease activity of sgRNA candidates We selected *AtDMP8*/*9* orthologues, *Glyma.18G097400* (*GmDMP8*) and *Glyma.18G098300* (*GmDMP9*), which have 97.6% identity and exhibit pollen-specific and pistil-abundant expression patterns, respectively. Through *in silico* prediction, a set of sgRNAs targeting the conserved sites of these genes was identified, and two sgRNAs were chosen for the *in vitro* cleavage assay (**Supplementary Table S7**). The assay demonstrated that both sgRNAs effectively guided Cas9 to cleave the target DNA sequences, resulting in the generation of specific fragment sizes. In the SgDMP#1 assay, 607 and 295 bp fragments were generated, while the SgDMP#2 assay produced fragments of 624 and 278 bp (**Figure 6**). Notably, based on intensity digestion of target PCR product, SgDMP#1 displayed higher cleavage efficiency compared to SgDMP2.

## 4 Discussion

With the availability of high-throughput technologies, genome datasets, and bioanalytical tools, soybean is receiving growing attention in genome-wide gene analyzes, identifying and characterizing multiple gene copies in each gene family. In this study, we mined the soybean genome for DUF679 family genes and characterized them using publicly available transcriptome datasets and various bioinformatics tools. A total of 14 *DMP* genes were identified in the soybean genome, which was in the range of other dicots and monocots (Kasaras and Kunze, 2010; Cyprys et al., 2019). The lengths of the identified DMP proteins ranged from 136 to 222 amino acids (**Table 1**). The instability index showed that eight DMP proteins are stable, whereas six are unstable. The majority of the *GmDMP*s had positive GRAVY index scores, suggesting that they might be membrane proteins, mainly interacting with hydrophobic regions that span membranes, rather than globular proteins. Peptides with fewer charged groups are generally less soluble in water and are disposed to aggregation in and interaction with hydrophobic pockets of larger proteins (Lawrence et al., 2007; Niwa et al., 2009).

In the phylogenetic analysis, seven major clades were formed. The monocot-/dicot-specific sub-clustering patterns corroborate previous studies, suggesting a common ancestor before the divergence of monocot and dicot *DMP* genes (Cyprys et al., 2019; Zhu et al., 2021). The pollen-specific *AtDMP8/9* orthologues (23 DMPs) included three soybean *DMPs* (*Glyma.09G237500*, *Glyma.18G097400*, and *Glyma.18G098300*). Orthologues of *AtDMP8/9* were utilized in a *DMP*-based haploid inducer system to efficiently induce maternal haploids in maize (Zhong et al., 2019), *Medicago truncatula* (Wang et al., 2022), *Brassica napus* (Zhong et al., 2022b), tobacco (Zhang et al., 2022b), and tomato (Zhong et al., 2022a). Our qPCR analysis revealed that among the *AtDMP8/9* orthologues, *Glyma.18G097400* and *Glyma.18G098300* exhibited pollen-specific and pistil-abundant expression patterns, respectively. While, *Glyma.09G237500* was highly expressed in reproductive organs, pollen and pistil, at the reproductive stage (**Figure 5**). This suggests their potential involvement in pollination and fertilization. A recent study demonstrated that the loss of function of the gynoecium-expressed phospholipase AII (*pPLAIIγ*) created haploid plants in dicotyledonous *Arabidopsis* (Jang et al., 2023), where the gynoecium-expressed *pPLAIIγ* induced female organ-mediated haploid induction. This highlights a potential of pistil-expressed *Glyma.09G237500* and *Glyma.18G098300* in female organ-mediated haploid induction, which could be further explored in future studies. So far, pollen-specific phospholipase A type of gene named *MTL/NLD/ZmPLA1* was reported to the functional maternal haploid inducer in monocotyledonous plants such as maize, rice, wheat, and foxtail millet (Gilles et al., 2017; Kelliher et al., 2017; Liu et al., 2017; Yao et al., 2018; Liu et al., 2020; Cheng et al., 2021). Thus, we suggest that *Glyma*.*18G097400* might be the potential target gene of maternal haploid inducer. Female organ-mediated haploid induction lines could be a promising tool as they allow the production of haploid plants without laborious emasculation procedures. Additionally, depending on germ lines, it may be more efficient than male-mediated haploid induction, as the female reproductive organ is the site of fertilization and zygote formation, providing a more direct route to haploid embryo production.

Subsequently, in an *in vitro* cleavage assay, we observed variations in the digestion efficiency of SgRNAs. SgRNA#1, targeting *Glyma.18G097400*, and *Glyma.18G098300*, exhibited higher cleavage efficiency compared to SgRNA#2, showing more cleaved bands from the originally amplified band size (**Figure 6**). Comparative studies, have found that RNP complexes capable of efficiently cleaving their target sites *in vitro* consistently produce similar results in protoplast-based screening methods (Kim et al., 2017; Jeong et al., 2019; Kim et al., 2020; Sant’Ana et al., 2020; Subburaj et al., 2022). Notably, the use of this *in vitro* cleavage protocol offers several advantages over alternative methods. All required components are commercially available or can be easily produced in the laboratory at a low cost (Mehravar et al., 2019; Bente et al., 2020). Additionally, the entire procedure can be completed within a single day. These findings highlight the effectiveness of the rapid, simple, and cost-effective *in vitro* cleavage protocol in eliminating inefficient candidate SgRNAs and identifying those with optimal performance, thereby increasing the likelihood of successful *in vivo* functions.

To elucidate the potential regulatory roles of *GmDMP*s in the development and stress response of soybean, we studied the distribution and frequency of CAREs. The identification of CAREs is currently an intriguing area, allowing the study of complex gene expression by integrating computational, comparative, and functional genomics (Li et al., 2015; Ho and Geisler, 2019; Nawade et al., 2022). The light-responsive elements (LREs) were found to be prevalent in the *GmDMP* promoters. Most common LREs, Box 4,G-box, and GT1-motif have been demonstrated to be critical for the regulation of light-mediated transcriptional activity (Gangappa et al., 2013; Ezer et al., 2017). The highly conserved G-box motif (CACGTG) binds to the basic helix-loop-helix (bHLH) and basic leucine zipper (bZIP) families of protein motifs (Heim et al., 2003; Carretero-Paulet et al., 2010) and reported to involved in the regulation of chlorophyll biosynthesis in *Arabidopsis* (Menkens et al., 1995; Kobayashi et al., 2012). The GATA motif (detected in 4 *GmDMP*s) plays a role in light responsiveness and tissue specificity, and is involved in the light-dependent development of phloem tissues (Trishla et al., 2020). In photosynthetic-responsive gene promoters, both I-box and G-box elements have been shown to be essential for activation in response to phytochrome, cryptochrome, and plastid signals. (Martıínez-Hernández et al., 2002; López-Ochoa et al., 2007). Twelve *GmDMP* promoters have either I-box or G-box, suggesting their functional relevance as regulators of ribulose 1,5-bisphosphate carboxylase/oxygenase small subunit light-responsive units (Murata et al., 2002). The promoter of *Glyma.18G098300* was found to have a site for the sp1 (GGGCGG) element (**Figure 3**, **Supplementary Table S5**), which is considered a mammalian promoter element, implicated in the regulation of a wide variety of housekeeping genes and tissue-specific genes (Hagen and Guilfoyle, 2002). Interestingly it was not found in the *Arabidopsis* or rice genomes (Yamamoto et al., 2007). The AAGAA-motif, activation sequence-1 (as-1; TGACG), O2-site, and GCN4_motif were the most frequently distributed CAREs related to plant growth and development (**Figure 3**, **Supplementary Table S5**). The as-1 element was found to be responsible for auxin- or salicylic acid-dependent enhanced expression in leaves (Niggeweg et al., 2000), whereas the AAGAA-motif was associated with secondary xylem development (Ain-Ali et al., 2021).

**Figure 7:**
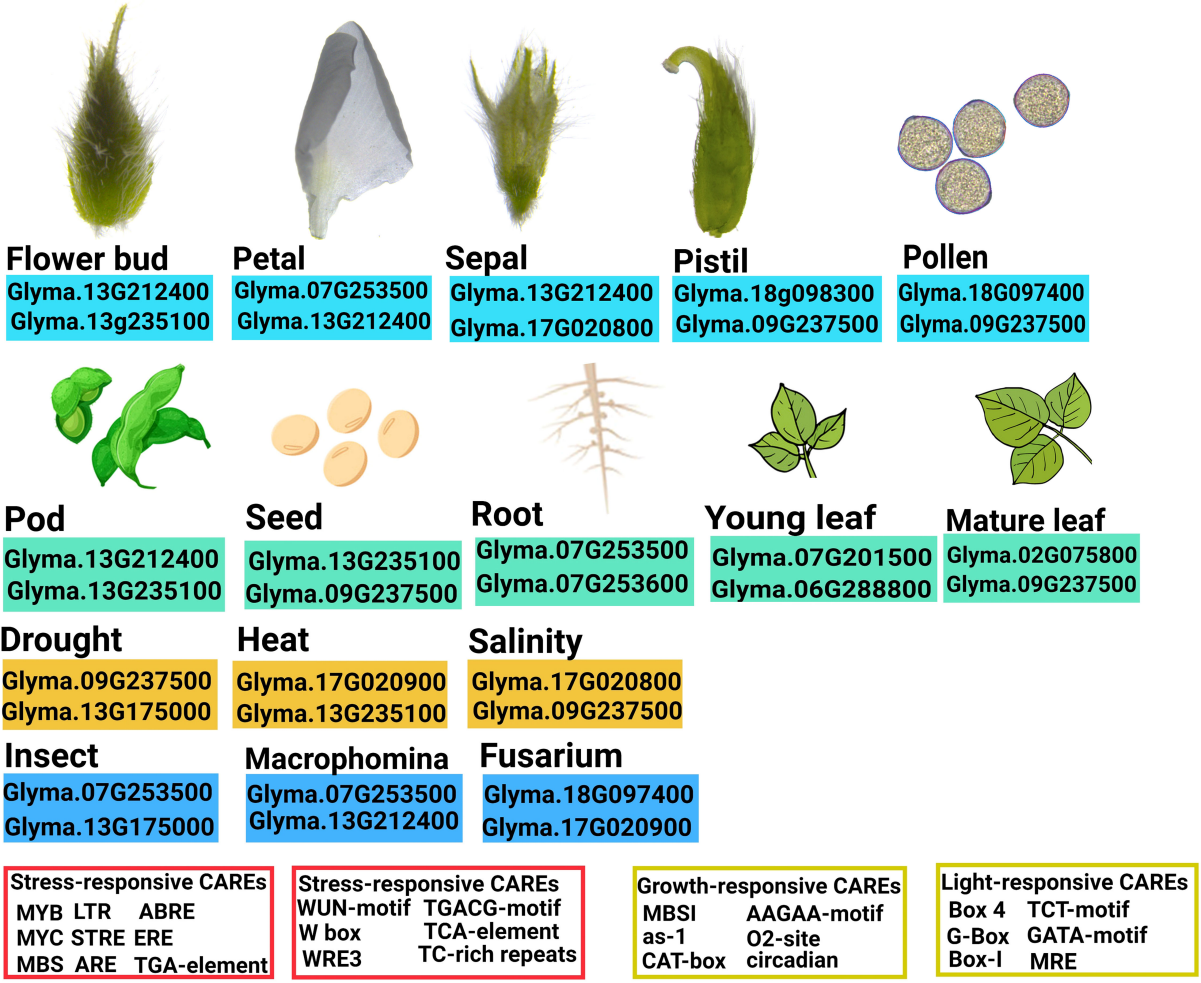
Overview of the expression profiles and key cis-acting regulatory elements of soybean *DMP* genes.

The promoters of soybean DMP genes have been also found to contain CAREs that are known to modulate gene expression in response to various stresses. Among abiotic stress-responsive CAREs, MYC, and MYB binding sites were present in all promoters. They have been reported to play an important role in drought-inducible expression, indicating that *GmDMP* expression is associated with abiotic stress (Smita et al., 2015). *Glyma.07G201500*, *Glyma.09G237500*, and *Glyma.07G253500*, which showed higher expression during drought, harbored multiple binding sites for dehydration-responsive elements (**Figure 3**, **Supplementary Table S5**). Moreover, the presence of LTR (low temperature responsive) in the *Glyma.16G157800* promoter suggests its involvement in cold stress response (Baker et al., 1994; Zhang et al., 2020). The ARE (anaerobic responsive elements) motifs, known as low oxygen and dehydration-induced elements (Dolferus et al., 2001), were found in 10 *GmDMP*s (**Supplementary Table S5**). The abscisic acid responsive elements (ABREs) have higher binding sites in most of the *GmDMP* promoters (**Figure 3**, **Supplementary Table S5**). They are regulators of various processes, including stomatal closure and seed and bud dormancy, as well as mediators in plant responses to cold, drought, and salinity stress (Choi et al., 2000; Yoshida et al., 2015). CAREs involved in the methyl-jasmonate responsive elements (TGACG-motif and CGTCA-motif) were also present in 10 *GmDMP* promoters (**Figure 3**, **Supplementary Table S5**). These elements are crucial in TF-mediated gene regulation (Rouster et al., 1997). For example, TGA1, a bZIP TF, was reported to act as a positive regulator of disease resistance by binding at the TGACG-motif and CGTCA-motif of the pathogenesis-related (*PR-1*) gene promoter in the *Arabidopsis* (Shearer et al., 2012). Likewise, the TGACG motif from the rice 12-oxophytodienoic acid reductase-1 (*OsOPR1*) promoter has been shown to play essential roles in defense responses (Sobajima et al., 2007). Moreover, the existence of W-box, WRE3, and the WUN-motif in promoters suggested that *GmDMP*s might play a vital role in biotic stress responses. The WUN-motif was characterized as wound responsive in the *WUN1* gene (Siebertz et al., 1989), while WRKY TFs are known to function in wound response by binding to W-boxes (Eulgem et al., 2000). Moreover, the presence of these CAREs and their elevated expression upon *Fusarium* infection suggest the possible role of *Glyma.18G097400* and *Glyma.18G098300* in fungal response (**Figure 4B**, **Supplementary Table S6**). Between the two *GmDMP*s in sub-clade III-1, *Glyma.16G157800* and *Glyma.02G075800*, *Glyma.02G075800* expression was 3-fold more elevated in *Macrophomina* resistant genotype. Notably, these *AtDMP10* orthologue harbors wounding and pathogen responsive CAREs (**Supplementary Table S5**). The W-box (TTGACC) element, which interacts with WRKY transcription factors (TFs) and regulates the expression of defense-related (*pathogenesis-related 10*, *PR-10*) genes, has a role in biotic and abiotic stresses, seed dormancy, and senescence (Dhatterwal et al., 2019). Under different biotic and abiotic stresses, the *DMP* genes exhibited diverse expression patterns and presence of stress responsive CAREs, indicating their potential roles in improving stress resistance and survival in soybean (**Figure 7**). However, further studies are required to validate the functional relevance of these genes during stress.

In summary, *DMP* genes retrieved from soybean were subjected to bioinformatics analyzes for characterization. In the phylogenetic analyzes, group V, composed of *AtDMP3* and *AtDMP5*, orthologues were absent from the soybean genome. The *AtDMP8/9* orthologues *Glyma.18G097400* and *Glyma.18G098300*, showing pollen-specific and pistil-abundant expression, could be potential targets for haploid induction in soybean. The expression profiles for most *GmDMP*s showed varying expression patterns in response to biotic and abiotic stresses. The presence of binding sites for various regulatory elements in *GmDMP* promoter sequences is consistent with this divergent expression pattern and implicates their possible involvement in growth and stress responses.

## Data availability statement

The datasets presented in this study can be found in online repositories. The names of the repository/repositories and accession number(s) can be found in the article/**Supplementary Material**.

## Author contributions

OL acquired the funds and resources, designed the experiment, and administered and supervised the project. OL, BN, and TB conceptualized the project. OL, BN, JL, and JJ designed the methodology, curated the data, and performed the formal analysis. TB performed the RNA-Seq analysis. BN and TB implemented the software and visualized and validated the data. OL, BN, TB, and JJ wrote the paper. All authors contributed to the article and approved the submitted version.
