# Supplementary material for: Genome-wide characterization of the soybean DOMAIN OF UNKNOWN FUNCTION 679 membrane protein gene family highlights their potential involvement in growth and stress response
Source: Front Plant Sci. 2023 Sep 8;14:1216082. doi: 10.3389/fpls.2023.1216082 (PMC10514519; doi:10.3389/fpls.2023.1216082)
Supplement: Supplementary file 1 [file DataSheet_1.docx]

Supplementary Material

Genome-wide characterization of soybean DOMAIN OF UNKNOWN FUNCTION 679 membrane protein (DMP) gene family highlights their potential involvement in growth and stress response.

**Bhagwat Nawade ^1,2†^, Tejas C. Bosamia^3†^, Jae Hyun Lee^1,2^, Jin Hoon Jang^1,2^, and Ok Ran Lee^1,2*^**

*** Correspondence:** Ok Ran Lee (mpizlee@jnu.ac.kr)


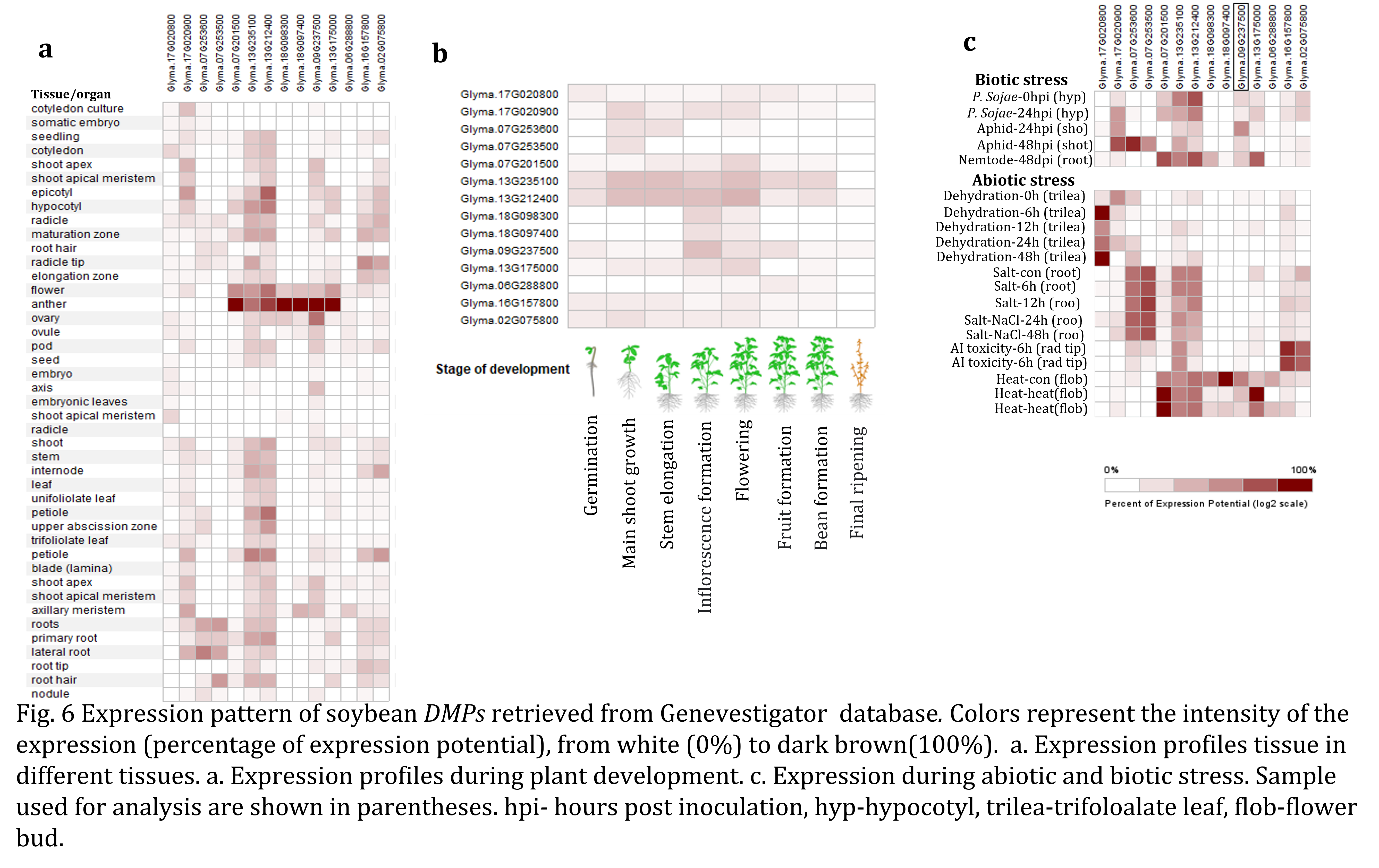


**Supplementary Figure S1.** Expression pattern of soybean *DMPs* retrieved from Genevestigator database*.* Colors represent the intensity of the expression (percentage of expression potential), from white (0%) to dark brown (100%). **a.** Expression profiles tissue in different tissues. **b.** Expression profiles during plant development. **c.** Expression during abiotic and biotic stress. Samples used for analysis are shown in parentheses. hpi*-* hours post-inoculation, hyp-hypocotyl, trilea-trifoloalate leaf, flob-flower bud.
